# Repair Rates for Multiple Descriptions on Distributed Storage †

**DOI:** 10.3390/e24050612

**Published:** 2022-04-27

**Authors:** Anders Høst-Madsen, Heecheol Yang, Minchul Kim, Jungwoo Lee

**Affiliations:** 1 Department of Electrical Engineering, University of Hawaii Manoa, Honolulu, HI 96822, USA; 2 Division of Computer Convergence, Chungnam National University, 99 Daehak-ro, Yuseong-gu, Daejeon 34134, Korea; hcyang@cnu.ac.kr; 3 Department of Electrical and Computer Engineering, Seoul National University, Seoul 08826, Korea; kmc1222@cml.snu.ac.kr (M.K.); junglee@snu.ac.kr (J.L.)

**Keywords:** distributed storage, multiple description coding, rate-distortion, lossy source coding, repair

## Abstract

In a traditional distributed storage system, a source can be restored perfectly when a certain subset of servers is contacted. The coding is independent of the contents of the source. This paper considers instead a lossy source coding version of this problem where the more servers that are contacted, the higher the quality of the restored source. An example could be video stored on distributed storage. In information theory, this is called the multiple description problem, where the distortion depends on the number of descriptions received. The problem considered in this paper is how to restore the system operation when one of the servers fail and a new server replaces it, that is, repair. The requirement is that the distortions in the restored system should be no more than in the original system. The question is how many extra bits are needed for repair. We find an achievable rate and show that this is optimal in certain cases. One conclusion is that it is necessary to design the multiple description codes with repair in mind; just using an existing multiple description code results in unnecessary high repair rates.

## 1. Introduction

In distributed storage systems, data is divided into multiple segments that are then stored on separate servers. In a typical setup [1], data is divided into *k* segments that are stored on *n* servers using an (n,k) maximum distance separable (MDS) code. If a user is able to contact any set of *k* servers, the data can be reconstructed. Notice that in this setup, if the user is able to contact less than *k* servers, it can retrieve no information, while on the other hand, there is no advantage in being able to contact more than *k* servers. One could instead want the *quality* of the reconstructed data to depend on how many servers a user is able to contact. An example could be video: it is common that the quality of streamed video depends on the network connection. In the context of distributed storage, the quality would now be dependent on the number of servers possible to connect, which could be constrained by network connection, physical location, delay, or cost. In information theory, this is known as multiple description coding [2,3]. Originally, multiple description coding was aimed at packet transmission networks, where some packets may be lost, but it can be directly applied to the distributed storage problem. We will accordingly call the systems we consider *multiple description distributed storage*.

A central issue in distributed storage is how to repair the system when one or more of the servers fail or become unavailable and are replaced by new servers [1]. In traditional distributed storage, this is also solved by the MDS code: if one server fails, the repair can be done by contacting *k* surviving servers, reconstruct the source, and then generating a new coded segment. The problem we consider in this paper is how repair can be done for multiple description distributed storage. The paper [1] and many following papers also consider how much network traffic is required for repair. However, in this paper we will only consider the *amount of additional data needed to be stored for repair to be possible*. The amount of network traffic is a topic for future research.

In general, the quality of reconstruction could be dependent not only on the number of servers connected, but which servers. However, to simplify the problem, we only consider the symmetric scenario where the quality only depends on the number of servers. This is the symmetric multiple description problem considered in [4]. A multiple description coding system with repair is specified as follows: when a subset J⊂{1,…,n} of servers is contacted, a source *X* should be restored with a distortion at most $D_{J}$. If one (or multiple) of the servers fails, we should be able to set up a replacement server with enough information so that the whole region $D_{J},J\subset \left\{1,\ldots ,n\right\}$ is restored. We consider two scenarios:

1. There is a special (highly reliable) repair server that does not participate in the usual operation of the system, but only comes into action if another server fails. The repair server can contact all other (non-failed) servers and use their information combined with its own information to restore the failed server (*collaborative repair*).
2. The repair information is stored in a distributed fashion among the *n* servers (*distributed repair*).

For simplicity, in this paper we only consider failure of a single server.

A straightforward solution is to separate the source coding problem (multiple description) and the repair problem. Any existing code for multiple description can then be used, and repair can be done using minimum distance separable (MDS) erasure codes as in traditional distributed storage [1]. We will use this as a baseline. For case 1 above, the repair server can simply store the xor (sum modulo 2) of the bits on the operational servers. When one server fails, the xor together with the bits from the surviving servers can restore the failed server. Thus, if each operational server stores lR bits, the repair server also needs to store lR bits. For distributed repair, the xor can replaced with an (n,n−1) erasure code. Therefore in addition to the lR bits for operation, each server needs to store $\frac{lR}{n-1}$ bits for repair. It should be clear that these rates are also optimal with separation: even if the system knows in advance which server will fail, it cannot store less information. We can consider this as a separate source channel coding solution, with multiple description being source coding and the repair being channel coding. It is known that in many information theory problems, joint source–channel coding is superior to separation. This is then the question we consider here: can we find a better joint source–channel coding solution that can beat the above rates? We will see that for some cases of desired distortion, separation is in fact optimal, while in other cases, joint source–channel coding provides much better rates.

The problem of repair of multiple description has been considered in some previous papers. In [5], the authors consider a problem like 1. above, but they do not give a single letter description of rate-distortion regions. In [6], the authors consider practical codes for repairing. In the current paper we aim to provide single letter expression for achievable rate-distortion regions, and in some cases the actual rate-distortion region. This paper is an extended version of our conference paper [7] with proof of the general achievable rate and specialization to the two level case, where we can prove optimality in certain cases.

## 2. Problem Description

In the following, we use the term *repair node* for the special repair server and *operational nodes* to denote the other servers. We let $I_{k}=\left\{1,\ldots ,k\right\}$ and $X_{I_{k}}=\left[X_{1},\ldots ,X_{k}\right]$, with the definition $I_{0}=\emptyset$ and $X_{I_{0}}=\left[\right]$ (e.g., $H\left(Y|X_{I_{0}}\right)=H\left(Y\right)$). For variables with multiple indices, $X_{I_{k},I_{j}}$ denotes a matrix of variables, i.e, the collection $\{X_{11},X_{12},\ldots ,X_{1j},X_{21},\ldots ,\ldots ,X_{k1},$$X_{k2},\ldots ,X_{kj}\}$, and $X_{kI_{j}}$ denotes a row.

We consider a *symmetric* multiple description problem as in [4]. We have an i.i.d. (independent identically distributed) source *X* that takes values in a finite alphabet X and needs to be restored in the finite alphabet $\hat{X}$; this can be generalized to a continuous alphabet Gaussian source through usual quantization arguments [3]. Let $J\subset I_{n}$. We are given a collection of distortion measures ${\tilde{d}}_{\left|J\right|}:X\times \hat{X}\to R^{+}$, and define

$$d_{\left|J\right|}\left(x^{l},{\hat{x}}^{l}\right)=\frac{1}{l}\sum_{i=1}^{l}{\tilde{d}}_{\left|J\right|}\left(x_{i},{\hat{x}}_{i}\right)$$

The required maximum distortion $D_{J}$ is then a function of |J| and the distortion measures $d_{\left|J\right|}$ only.

### 2.1. Distributed Repair

We will first define the distributed repair problem. For a source sequence $x^{l}$ of length *l*, each node stores $lR_{t}$ bits. There are *n* encoding functions $f_{i}:X^{l}\to \left\{1,\ldots ,2^{lR_{t}}\right\}$, $2^{n-1}-1$ decoding functions $g_{J}:{\left\{1,\ldots ,2^{lR_{t}}\right\}}^{\left|J\right|}\to {\hat{X}}^{l}$, $J\subset I_{n},1\leq \left|J\right|\leq n-1$, and *n* repair functions $h_{i}:{\left\{1,\ldots ,2^{lR_{t}}\right\}}^{n-1}\to \left\{1,\ldots ,2^{lR_{t}}\right\}$. We define the error probability of repair as

$$P_{r}^{\left(l\right)}=\max_{i=1,\ldots ,n}P\left(h_{i}\left(f_{I_{n}-\left\{i\right\}}\left(x^{l}\right)\right)\neq f_{i}\left(x^{l}\right)\right).$$

Here, $f_{I_{n}-\left\{i\right\}}\left(x^{l}\right)$ is the length n−1 list obtained by removing the *i*-th component from $(f_{1}\left(\left(x^{l}\right),f_{2}\left(x^{l}\right),\ldots ,f_{n}\left(x^{l}\right)\right)$. We now say that an a tuple $\left(R_{t},D_{1},\ldots ,D_{n-1}\right)$ is achievable if there exists a sequence of $\left(2^{lR_{t}},l\right)$ codes with

(1) $$\begin{array}{cc} \forall m<n:\lim_{l\to \infty }\max_{J:|J|=m}E\left[d_{\left|J\right|}\left(x^{l},g_{J}\left(f_{J}\left(x^{l}\right)\right)\right)\right] & \leq D_{m}\\ \lim_{l\to \infty }P_{r}^{\left(l\right)} & =0 \end{array}$$

We call this *exact repair*. The repaired node is required to be an exact copy of the failed node, except that we allow a certain, vanishing, and error rate. Notice that the randomness in the system is purely due to the source $x^{l}$. Thus, for a given sequence $x^{l}$, either all failures can be repaired exactly, and if they can be repaired once, they can be repaired infinitely many times; or, some failures can never be repaired. The probability of the source sequences that are not repairable should be vanishingly small.

An alternative problem formulation, which we call *functional repair*, is to allow approximate repair, where the only requirement is that after repair the distortion constraint is satisfied. In that case, one would have to carefully consider repeated repair. In this paper, we will only consider exact repair for coding schemes. It should be noted that in the cases where we have tight converses (the two node case [7], Theorem 3 in some scenarios), the converses are actually for functional repair; thus, functional repair might not decrease rates.

### 2.2. Collaborate Repair

For collaborate repair with a dedicated repair node, each node stores lR bits and the repair node $lR_{r}$ bits. There are now *n* encoding functions $f_{i}:X^{l}\to \left\{1,\ldots ,2^{lR}\right\}$ and additionally a repair encoder $f_{r}:X^{l}\to \left\{1,\ldots ,2^{lR_{r}}\right\}$, $2^{n}-1$ decoding functions $g_{J}:{\left\{1,\ldots ,2^{lR_{t}}\right\}}^{\left|J\right|}\to {\hat{X}}^{l}$, $J\subset I_{n},1\leq \left|J\right|\leq n$, and *n* repair functions $h_{i}:{\left\{1,\ldots ,2^{lR}\right\}}^{n-1}\times \left\{1,\ldots ,2^{lR_{r}}\right\}\to \left\{1,\ldots ,2^{lR}\right\}$. We define the error probability of repair as

$$P_{r}^{\left(l\right)}=\max_{i=1,\ldots ,n}P\left(h_{i}\left(f_{I_{n}-\left\{i\right\}}\left(x^{l}\right),f_{r}\left(x^{l}\right)\right)\neq f_{i}\left(x^{l}\right)\right)$$

We now say that an a tuple $\left(R,R_{r},D_{1},\ldots ,D_{n}\right)$ is achievable if there exists a sequence of $\left(2^{lR},2^{lR_{r}},l\right)$ codes with

(2) $$\begin{array}{cc} \forall m\leq n:\lim_{l\to \infty }\max_{J:|J|=m}E\left[d_{\left|J\right|}\left(x^{l},g_{J}\left(f_{J}\left(x^{l}\right)\right)\right)\right] & \leq D_{m}\\ \lim_{l\to \infty }P_{r}^{\left(l\right)} & =0 \end{array}$$

## 3. Achievable Rate

The rate-distortion region for multiple description coding is only known in a few cases; among those are the two node Gaussian case first studied in [2], and the two level case studied in [8,9]. There are, therefore, many different achievable schemes for multiple description coding, e.g., [4,10,11,12], and we have to design repairs for each specific method. In this paper, we will consider the Puri Pradhan Ramchandran (PPR) scheme [4,13], as this is specifically aimed at the symmetric case and is well-suited for repair. It is optimal in certain cases [8,9], but not always [11].

The coding method in [4] is based on source-channel erasure codes (SCEC) from [13]. An (n,k)-SCEC is similar to an (n,k)-MDS erasure code: if any *k* of *n* packets are received, the transmitted message can be recovered with a certain distortion. However, with an (n,k)-SCEC if m>k packets are received, the message can be recovered with decreasing distortion with *m*. Using a concatenation of (n,1),(n,2),…,(n,n) SCEC, [4] obtained the following result

**Proposition** **1** (PPR [4]). *For any symmetric probability distribution $p(y_{I_{n-1},I_{n}},y_{n}|x)$ the lower convex closure of $\left(R,D_{1},\ldots ,D_{n}\right)$ is achievable, where $E[d_{\left|J\right|}\left(X,g_{J}\left(Y_{I_{\left|J\right|}J}\right)\right]\leq D_{\left|J\right|},|J|\leq n$ and*

$$\begin{array}{cc} R & \geq \sum_{k=1}^{n-1}\frac{1}{k}H\left(Y_{kI_{k}}|Y_{I_{k-1},I_{k}}\right)\\ \; & +\frac{1}{n}I\left(Y_{n};X|Y_{I_{n-1}I_{n}}\right)-\frac{1}{n}H\left(Y_{I_{n-1}I_{n}},Y_{n}|X\right) \end{array}$$

A probability distribution $p(y_{I_{n-1},I_{n}},y_{n-1}|x)$ is symmetric if for all $1\leq r_{i}\leq n,i\in I_{n-}$ the joint distribution of $Y_{n-1}$ and all $\left(r_{1}+r_{2}+\cdots +r_{n-1}\right)$ random variables where any $r_{i}$ are chosen from the *i*th layer, conditioned on *X* are the same.

We first notice that for collaborative repair, reconstruction from *n* nodes does not make sense: since we can repair the last node from n−1 nodes, there can be no gain for a user to access all *n* nodes. The performance is therefore specified by $\left(D_{1},D_{2},\ldots ,D_{n-1}\right)$. As a baseline, we thus consider the standard PPR scheme where we use at most n−1 nodes for the reconstruction. Now, in layer n−1, we just need a single common message (in standard PPR that happens at layer *n*). This message can be encoded using an (n,n−1) MDS erasure code. We then get the following rate, which we state without proof as it is a simple modification of PPR:

**Proposition** **2.** *For any symmetric probability distribution $p(y_{I_{n-2},I_{n}},y_{n-1}|x)$ the lower convex closure of $\left(R,D_{1},\ldots ,D_{n-1}\right)$ is achievable, where $E[d_{\left|J\right|}\left(X,g_{J}\left(Y_{I_{\left|J\right|}J}\right)\right]\leq D_{\left|J\right|},|J|\leq n-1$, the following rate is achievable with n nodes and using at most (n−1) nodes for reconstruction*

$$\begin{array}{cc} R & \geq \sum_{k=1}^{n-2}\frac{1}{k}H\left(Y_{kI_{k}}|Y_{I_{k-1},I_{k}}\right)\\ \; & +\frac{1}{n-1}I\left(Y_{n-1};X|Y_{I_{n-2}I_{n-1}}\right)-\frac{1}{n}H\left(Y_{I_{n-2}I_{n}}|X\right) \end{array}$$

Notice that one should not think of this as an ‘improved’ PPR scheme; rather it is the PPR scheme adapted to the special case here, where at most n−1 nodes are used for reconstruction.

**Figure 1:**
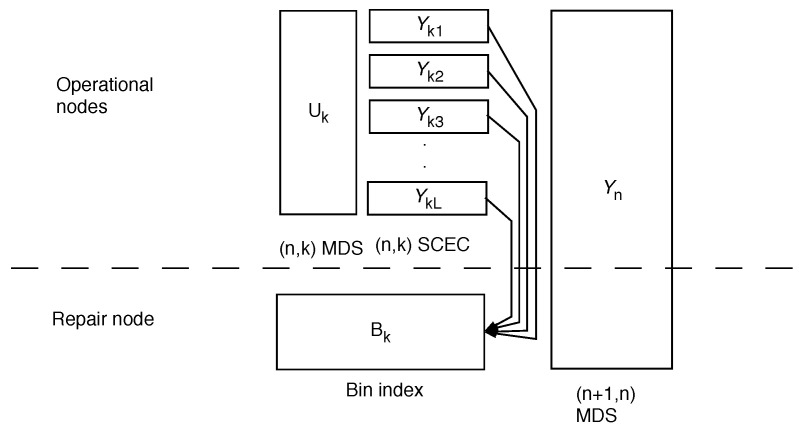
Two layer repair. See text for explanation.

For our repair coding scheme, we amend the PPR scheme, specifically from Proposition 2. We still use an (n,k)-SCEC at layers k≤n−2, but add a common message ($U_{k}$) at each layer k≤n−2. At layer 1, this is a true common message that is duplicated to all nodes. At layers k>1 this is a message stored with an (n,k)-MDS code. Common messages were shown to be necessary to achieve optimality for the two-node case in [7]. We also use binning for repair of correlated quantizations. A system schematic for a specific case can be seen in Figure 1 below. The addition of common messages strictly decreases the rate for repair in some cases, see Section 5.

The following is the main result of the paper, an achievable repair rate; this rate can be compared to the rate in Proposition 2. As above, we call a probability distribution $p(y_{I_{n-2},I_{n}},u_{I_{n-2}},y_{n-1}|x)$ symmetric if for all $1\leq r_{i}\leq n-1,i\in I_{n-2}$ and all $k\in I_{n-2}$ the joint distribution of $Y_{n-1},U_{k}$ and all $\left(r_{1}+r_{2}+\cdots +r_{n-2}\right)$ random variables where any $r_{i}$ are chosen from the *i*th layer, conditioned on *X* are the same.

**Theorem** **1** (Distributed repair). *For any symmetric probability distribution $p(y_{I_{n-2},I_{n}},u_{I_{n-2}},$$y_{n-1}\left|x\right)$ the lower convex closure of $\left(R+R_{r},D_{1},\ldots ,D_{n-1}\right)$ is achievable, where $E[d_{\left|J\right|}(X,g_{J}(Y_{I_{\left|J\right|}J},$$U_{I_{\left|J\right|}})]\leq D_{\left|J\right|},\left|J\right|\leq n-1$ and the information needed to encode operational information is*

$$\begin{array}{c} R>\sum_{k=1}^{n-2}\frac{1}{k}H\left(Y_{kI_{k}}|U_{I_{k}},Y_{I_{k-1}I_{k}}\right)-\frac{1}{n}H\left(Y_{kI_{n}}|X,U_{I_{k}},Y_{I_{k-1}I_{n}}\right)\\ +\frac{1}{n-1}I\left(Y_{n-1};X|Y_{I_{n-2}I_{n-1}},U_{I_{n-2}}\right)\\ +\sum_{k=1}^{n-2}\frac{1}{k}\left(H\left(U_{k}|Y_{I_{k-1}I_{k}},U_{I_{k-1}}\right)-H\left(U_{k}|X,Y_{I_{k-1}I_{n}},U_{I_{k-1}}\right)\right) \end{array}$$

*with additional information needed to encode repair information*

Rr>1n−1∑k=1n−2H(Ykn|UIk,YkIn−1YIk−1In)−1nH(YkIn|X,Yk−1In,UIk)−1nH(YkIn|X,Yk−1In,UIk)+

*with ${\left[x\right]}^{+}=\max\left\{0,x\right\}$*

**Proof.**  There is a formal proof in Appendix A—the purpose here is to outline how the coding is done and how the rate expressions are obtained, without a deep knowledge of [4].

Consider at first layer 1. We generate a codebook $C_{u1}$ by picking $2^{lR_{u1}^{\prime }}$ elements uniformly randomly with replacement from the typical set according to the distribution $p_{U_{1}}\left(u_{1}\right)$. We also generate *n independent* random codebooks $C_{1I_{n}}$ drawn from the typical set according to $p_{Y_{11}}\left(y_{11}\right)$ with $2^{lR_{1}^{\prime }}$ codewords. We need to be able to find a codeword in $C_{u1}$ that is jointly typical with $x^{l}$ with high probability, which, from standard rate distortion, is the case if

$$\begin{array}{cc} R_{u1}=R_{u1}^{\prime } & >H\left(U_{1}\right)-H\left(U_{1}|X\right)=I\left(X;U_{1}\right) \end{array}$$

This codeword is stored in all the nodes. We now need to be able to find *n* codewords from $C_{1I_{n}}$ that are *jointly* typical with $x^{l}$ *and* the chosen codeword $u_{1}^{l}\in C_{u1}$. There are about $2^{nlH(Y_{11})}$ (marginally) typical sequences, and about $2^{lH(Y_{11},\ldots ,Y_{1n}|U_{1},X)}$ that are jointly typical with a given $x^{l}$ and $u_{1}^{l}$ (see, e.g., [14] (Section 15.2)); the probability that a given codeword combination in $C_{1I_{n}}$ is jointly typical, therefore it is about $2^{l(H\left(Y_{11},\ldots ,Y_{1n}|U_{1},X\right)-nH\left(Y_{11}\right))}$. The probability that *no* codeword is jointly typical then is about

$${\left(1-2^{l(H\left(Y_{11},\ldots ,Y_{1n}|U_{1},X\right)-nH\left(Y_{11}\right))}\right)}^{2^{nlR_{1}^{\prime }}}\leq \exp\left(-2^{l(nR_{1}^{\prime }-\left(nH\left(Y_{11}\right)-H\left(Y_{11},\ldots ,Y_{1n}|U_{1},X\right)\right))}\right)$$

The inequality is standard in rate distortion, see [3,14]. Thus, if

(3) $$\begin{array}{cc} nR_{1}^{\prime } & >nH\left(Y_{11}\right)-H\left(Y_{11},\ldots ,Y_{1n}|U_{1},X\right) \end{array}$$

there is a high probability that at least one of the $2^{nlR_{1}^{\prime }}$ codeword combinations is jointly typical.

The codewords in $C_{1j}$ are randomly binned into $2^{lR_{1}}$ bins. At the time of decoding, the common codeword $u_{1}^{l}\in C_{u1}$ is available as well as the bin number *i* for the codeword $y_{ij}^{l}\in C_{1j}$. The decoder looks for a codeword in bin *i* that is typical with $u_{1}^{l}$. There is always one, the actual codeword, but if there is more than one, the decoding results in error. The probability that a random codeword in $C_{1j}$ is jointly typical with $u_{1}^{l}$ is about $2^{l(H\left(Y_{11}|U_{1}\right)-H\left(Y_{11}\right))}$ as above, while there are about $2^{l(R_{1}^{\prime }-R_{1})}$ codewords in each bin. By the union bound, the probability that there is at least one random codeword in the bin jointly typical is approximately upper bounded by $2^{l(R_{1}^{\prime }-R_{1})}2^{-l(H\left(Y_{11}\right)-H\left(Y_{11}|U_{1}\right))}$. Thus, if

(4) $$R_{1}^{\prime }-R_{1}<H\left(Y_{11}\right)-H\left(Y_{11}|U_{1}\right)$$

there is only one such codeword with high probability. Combining (3) and (4) we get

$$R_{1}>H\left(Y_{11}|U_{1}\right)-\frac{1}{n}H\left(Y_{i1},\ldots ,Y_{in}|U_{1},X\right)$$

At layer k<n−1 we similarly generate a random codebook $C_{uk}$ with $2^{lR_{uk}^{\prime }}$ typical elements according to the marginal distribution $p_{U_{k}}\left(u_{k}\right)$ and *n* independent random codebooks $C_{kI_{n}}$ according to the distribution $p_{Y_{k1}}\left(y_{k1}\right)$ with $2^{lR_{k}^{\prime }}$ codewords. We need to be able to find a codeword in $C_{uk}$ that is jointly typical with $x^{l}$ and all the codewords chosen in the previous layers. This is possible if

$$\begin{array}{cc} R_{uk}^{\prime } & >H\left(U_{k}\right)-H\left(U_{k}|X,Y_{I_{k-1}I_{n}},U_{I_{k-1}}\right) \end{array}$$

with the same argument as for (3). We also need to be able to find an *n*-tuple of codewords from $C_{kI_{n}}$ that are jointly typical with all prior codewords and $x^{l}$, which is possible with high probability if (again as in (3))

$$\begin{array}{cc} nR_{k}^{\prime } & >nH\left(Y_{k1}\right)-H\left(Y_{kI_{n}}|X,Y_{k-1I_{n}},U_{I_{k}}\right) \end{array}$$

For $C_{uk}$, we generate *n* *independent* binning partitions each with $2^{lR_{uk}}$ elements. The bin number in the *i*-th partition is stored in the *i*-th node. When the decoder has access to *k* nodes, say nodes 1,…,k it needs to be able to be able to find a *unique* codeword in the *k* bins jointly typical with codewords from previous layers. The probability that a random selected codeword is jointly typical is about $2^{l(H\left(U_{k}|Y_{I_{k-1}I_{k}},U_{I_{k-1}}\right)-H\left(U_{k}\right))}$, as above. There are about $2^{lR_{uk}^{\prime }}2^{-lkR_{uk}}$ in each combined bin. Therefore, if

$$kR_{uk}>R_{uk}^{\prime }+H\left(U_{k}|Y_{I_{k-1}I_{k}},U_{I_{k-1}}\right)-H\left(U_{k}\right)$$

or

(5) $$R_{uk}>\frac{1}{k}\left(H\left(U_{k}|Y_{I_{k-1}I_{k}},U_{I_{k-1}}\right)-H\left(U_{k}|X,Y_{I_{k-1}I_{n}},U_{I_{k-1}}\right)\right)$$

with high probability there is only one jointly typical codeword in the combined bin. It also needs to find a single codeword in the *k* bins for $C_{kI_{k}}$ s that are jointly typical with $\left(U_{I_{k}},Y_{I_{k-1}I_{k}}\right)$. The probability that a random codeword is jointly typical is about $2^{l(H\left(Y_{kI_{k}}|U_{I_{k}},Y_{I_{k-1}I_{k}}\right)-kH\left(Y_{k1}\right))}$, while the number of codewords in the *k* joint bins is about $2^{lkR_{k}^{\prime }}2^{-lkR_{k}}$. With high probability there is only one such if

$$k\left(R_{k}^{\prime }-R_{k}\right)<kH\left(Y_{k1}\right)-H\left(Y_{kI_{k}}|U_{I_{k}},Y_{I_{k-1}I_{k}}\right)$$

or

$$R_{k}>\frac{1}{k}H\left(Y_{kI_{k}}|U_{I_{k}},Y_{I_{k-1}I_{k}}\right)-\frac{1}{n}H\left(Y_{kI_{n}}|X,U_{I_{k}},Y_{I_{k-1}I_{n}}\right)$$

(as in [13] this can be repeated for any collection of *k* nodes).

At layer n−1 only a single codebook is generated, and this is binned into *n* independent partitions. Upon receipt, in analogy with (5), this can be found uniquely with high probability if

$$\begin{array}{cc} R_{n-1} & >\frac{1}{n-1}H\left(Y_{n-1}|Y_{I_{n-2}I_{n-1}},U_{I_{n-2}}\right)\\ \; & -\frac{1}{n-1}H\left(Y_{n-1}|X,Y_{I_{n-2}I_{n}},U_{I_{n-2}}\right) \end{array}$$

For repair, the joint $2^{nlR_{k}^{\prime }}$ codewords in $C_{k1}\times \cdots \times C_{kn}$ at layer k<n−1 are binned into $2^{lR_{rk}}$ bins. The single bin number of the *n* chosen codewords is encoded with an (n,n−1) MDS erasure code.

Now, suppose node *n* is lost, and needs to be recovered. The repair node works from the bottom up. So, suppose the previous k−1 layers have been recovered, that is, $y_{I_{k-1}I_{b}}^{l},u_{I_{k-1}}^{l}$ are known without error. First $u_{k}^{l}$ is recovered, which can be done since n−1≥k nodes are used. It can also decode the codewords in $C_{kI_{n-1}}$. It restores the bin number of the repair codeword from the erasure code. There are approximately $2^{l(nR_{k}^{\prime }-R_{rk})}$ codewords in the bin, but since it knows the codewords in $C_{kI_{n-1}}$, there are only about $2^{l(R_{k}^{\prime }-R_{rk})}$ valid ones. It searches in the bin for valid codewords jointly typical with $y_{kI_{n-1}}^{l},y_{I_{k-1}I_{n}}^{l},u_{I_{k}}^{l}$. With high probability, there is only one such if

$$R_{k}^{\prime }-R_{rk}<H\left(Y_{kn}\right)-H\left(Y_{kn}|U_{I_{k}},Y_{kI_{n-1}}Y_{I_{k-1}I_{n}}\right)$$

(The right hand side could be negative. This means that the lost codeword can be recovered from the surviving ones without extra repair information. Then we just put $R_{rk}=0$.) Then

(6) $$\begin{array}{cc} R_{rk} & >H(Y_{kn}|U_{I_{k}},Y_{kI_{n-1}}Y_{I_{k-1}I_{n}})\\ \; & -\frac{1}{n}H\left(Y_{kI_{n}}|X,Y_{k-1I_{n}},U_{I_{k}}\right) \end{array}$$

There is at least one codeword in the bin, namely the correct one. Thus, if there is no error (more than one codeword), the repair is exact, as required from the exact repairability condition in Section 2. □

The above result can easily be adapted to the case of a repair node that collaborates with the operational nodes. There are only two differences:

- The repair node can restore operation of the full *n* node distortion region. Therefore, the terminal single common codeword is not at layer n−1, but at layer *n*. At the same time, the repair node now has to store repair information for this last codeword.
- For distributed repair, distributions are chosen to minimize $R+R_{r}$. For collaborative repair, distributions are chosen to minimize *R*, and $R_{r}$ is then as given for those distributions.

With this in mind, we get

**Theorem** **2** (Collaborative repair). *For any symmetric probability distribution $p(y_{I_{n-1},I_{n}},u_{I_{n-1}},$$y_{n}\left|x\right)$ the lower convex closure of $\left(R,D_{1},\ldots ,D_{n}\right)$ is achievable, where $E[d_{\left|J\right|}\left(X,g_{J}\left(Y_{I_{\left|J\right|}J},U_{I_{\left|J\right|}}\right)\right]\leq D_{\left|J\right|},|J|\leq n$ and*

$$\begin{array}{c} R>\sum_{k=1}^{n-1}\frac{1}{k}H\left(Y_{kI_{k}}|U_{I_{k}},Y_{I_{k-1}I_{k}}\right)-\frac{1}{n}H\left(Y_{kI_{n}}|X,U_{I_{k}},Y_{I_{k-1}I_{n}}\right)\\ +\frac{1}{n}I\left(Y_{n};X|Y_{I_{n-1}I_{n}},U_{I_{n-1}}\right)\\ +\sum_{k=1}^{n-1}\frac{1}{k}\left(H\left(U_{k}|Y_{I_{k-1}I_{k}},U_{I_{k-1}}\right)-H\left(U_{k}|X,Y_{I_{k-1}I_{n}},U_{I_{k-1}}\right)\right) \end{array}$$

*The additional information the repair node has to store is*

Rr>∑k=1n−1H(Ykn|UIk,YkIn−1YIk−1In)−1nH(YkIn|X,Yk−1In,UIk)−1nH(YkIn|X,Yk−1In,UIk)++1nH(Yn|YIn−1In,UIn−1)−1nH(Yn|YIn−1In,UIn−1,X)

The proof is nearly identical to the proof of Theorem 1, so it will be omitted.

## 4. The Two Level Case

In [9], the authors considered the situation when there were only two cases of node access: Either we have access to all *n* nodes, or we have access to a given number k<n nodes; there are two levels of distortion: $\left(D_{k},D_{n}\right)$. Importantly, they were able to derive the *exact* capacity region for this case for Gaussian sources, one of the few cases when this known except for the original EC case [2]. This makes it an interesting case to consider for repair: at least we can upper bound the number of bits needed for repair by the achievable rate in Section 3. The paper [9] considered the vector Gaussian case, but we restrict ourselves to the scalar Gaussian case.

To fit into the framework of [9], we need to consider the case when there is a repair node, Theorem 2. In that case, the scheme is as shown on Figure 1. The $U_{k}$ represents a common codeword that is stored jointly on the operational nodes with an (n,k) MDS. If one server fails, this can be restored without additional information from the repair as k≤n−1. $Y_{k1},\ldots ,Y_{kL}$ represent individual codewords using SCEC (source-channel erasure code) codes from [4,13]; here, the repair is accomplished using correlation and a bin index, similar to the two node case. Finally, $Y_{n}$ represents resolution information, which can be repaired due to the (n+1,n) MDS code.

The explicit rate constraints from Theorem 2 are

$$\begin{array}{c} R>\frac{1}{k}H\left(Y_{kI_{k}}|U_{k}\right)\\ +\frac{1}{n}H\left(Y_{n}|Y_{kI_{n}},U_{k}\right)-\frac{1}{n}H\left(Y_{kI_{n}},Y_{n}|X,U_{k}\right)\\ +\frac{1}{k}\left(H\left(U_{k}\right)-H\left(U_{k}|X\right)\right) \end{array}$$

with

$$\begin{array}{cc} R_{r} & >{\left[H\left(Y_{kn}|U_{k},Y_{kI_{n-1}}\right)-\frac{1}{n}H\left(Y_{kI_{n}}|X,U_{k}\right)\right]}^{+}\\ \; & +\frac{1}{n}H\left(Y_{n}|Y_{kI_{n}},U_{k}\right)-\frac{1}{n}H\left(Y_{n}|Y_{kI_{n}},U_{k},X\right) \end{array}$$

We consider an iid Gaussian source with $x_{i}∼N\left(0,1\right)$ with a quadratic distortion function: ${\tilde{d}}_{\left|J\right|}\left(x_{i},{\hat{x}}_{i}\right)={\left(x_{i}-{\hat{x}}_{i}\right)}^{2}$. For this situation, we can calculate the achievable repair rate explicitly. We recall that the problem setup is that *R* is fixed to the optimum rate from [9]. We then obtain:

**Theorem** **3.** *In the Gaussian two level case, we have the following bounds on the repair rate:*

*1.*  *For $k{\left(D_{k}^{-1}-1\right)}^{-1}-n{\left(D_{n}^{-1}-1\right)}^{-1}\leq 0$ a common message is used and achieves*
$$R_{r}\leq \frac{1}{2}\log\left(\frac{D_{k}\left(n-k\right)}{D_{k}\left(n-k-1\right)+D_{n}}\right)$$
*For k=n−1 the upper bound is tight.*
*2.*  *For $0<k{\left(D_{k}^{-1}-1\right)}^{-1}-n{\left(D_{n}^{-1}-1\right)}^{-1}\leq n-k$ no common message is used and*
$$R_{r}\leq \frac{1}{2}\log\left(\frac{\left(D_{k}-1\right)n\left(n-k\right){\left(\frac{k(D_{k}-D_{n})}{\left(D_{k}-1\right)D_{n}\left(k-n\right)}\right)}^{1/n}}{k\left(-D_{k}n+D_{n}+n-1\right)+\left(D_{k}-1\right)\left(n-1\right)n}\right)$$
*For k=n−1 the upper bound is tight.*
*3.*  *For $k{\left(D_{k}^{-1}-1\right)}^{-1}-n{\left(D_{n}^{-1}-1\right)}^{-1}>n-k$ no common message is used and the* exact repair rate *is*
  $$R_{r}=R=\frac{1}{2n}\log\left(\frac{1}{D_{n}}\right)$$
  *for all k and n.*

We will discuss some implications of this result. The converse is provided by the bound (A8) $\left(n-1\right)R+R_{r}\geq \frac{1}{2}\log\left(\frac{1}{D_{n}}\right)$, which is simply the requirement that the repair node together with the surviving nodes should be able to restore the source with distortion $D_{n}$. This is clearly also a converse for functional repair, which could indicate that relaxing to functional repair cannot decrease rates. For k=n−1, the theorem provides the exact repair rate; without using common messages, we could not have achieved the bound. We can compare with separate repair and multiple description coding, as mentioned in the introduction. For case 3, the theorem separation is optimal, but for the other cases $R_{r}<R$. For example, for $n=10,k=5,D_{k}=0.5,D_{n}=0.48$, we get $R=0.06,R_{r}=0.02$ for case 1.

## 5. Example Gaussian Case

**Figure 2:**
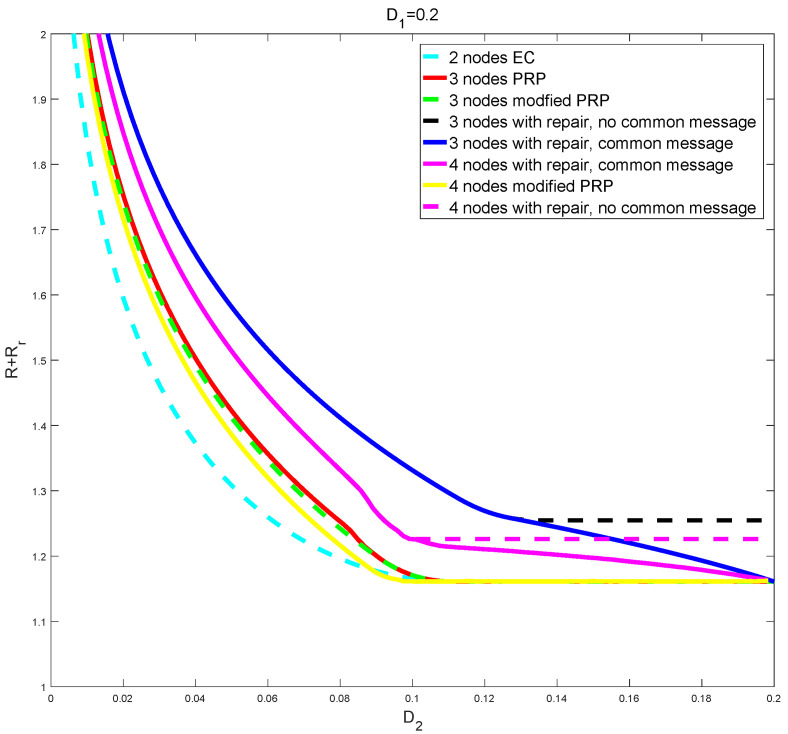
Plots of *R* or $R+R_{r}$ for two levels of constraints $\left(D_{1},D_{2}\right)$ and variable number of nodes.

Figure 2 shows typical numerical results. All curves are for two levels of constraints, $\left(D_{1},D_{2}\right)$, but variable number of nodes. First, from the bottom, we have the curve for the optimum region for the two node problem according to EC [2,3]. Notice that this is achieved without any refinement information, using only correlation between the base layer random variables; refinement information is only required for $D_{1}>\frac{1}{2}$ and $D_{2}<2D_{1}-1$. Second, we have the curves for the three node problem, but where we use at most two nodes for reconstruction, either using [4] (Section V) directly (ignoring the $D_{3}$ constraint), or using Theorem 1 without repair. It can be noticed that using Proposition 2 gives a slight improvement; this is *not* due to the common message, but due to the fact that PPR uses n−1 codewords in the last layer, while the modified PPR uses only one. For the 4 node case, we use (4,1)-SCEC and (4,2)-SCEC successively, as well as (4,1)-MDS common message and (4,2)-MDS common message. Therefore, we have 2 variables $U_{1}$ and $U_{2}$ for common messages, and $Y_{1i}$ and $Y_{2i}$ for SCEC, where *i* = 1, 2, 3, 4. As a result, it is noted that the overall rate of the 4 node system improves over that of the 3 node system, whereas the overall rate of the 2 node system improves over that of the 3 node system where common message and SCEC were used only once. We see that a common message gives a clear improvement.

## 6. Conclusions

The paper has derived achievable rates for repair of multiple description distributed storage, which in some cases is optimal. Our solution shows that joint repair and multiple description coding beats separate coding in many cases. It also shows that it is sub-optimal for repair to just take a standard multiple description code and add repair information. Rather, the multiple description code has to be designed with repair in mind. In this paper, we do this by adding common messages.

This paper is only a first step in solving repair of multiple description distributed storage. For one thing, we have assumed that the repair bandwidth is unlimited. When the required repair bandwidth is also of concern as in [1], an entirely new set of constraints comes into play. We will consider this in a later paper.

## Data Availability

Not applicable.

## Abbreviations

The following abbreviations are used in this manuscript:

MDS	Maximum distance separable (code)
EC	El-Gamal Cover (coding scheme)
ZB	Zhang-Berger (coding scheme)
PPR	Puri Pradhan Ramchandran (coding scheme)
SCEC	Source-channel erasure code

## Appendix A. Proof of Theorem 1

Contrary to the proof outline, which is intended to stand by itself, the formal proof is a modification of the proof of Theorem 2 in [4], and reading it requires a good familiarity with [4]. We will not repeat the proof in [4], but only the new elements. The proof in this paper adds common messages, which require a separate codebook generation, and an analysis of additional error events. It also adds repair codebooks, and an analysis of repair error.

We let $T_{\epsilon }^{l}\left(X\right)$ denote the strongly ϵ typical set for *X*.

The coding scheme for repair uses MDS codes in several places. These can be put in the binning framework of PPR [13]. However, it is easier to think of them as pure channel codes. We can state this as follows:

**Remark** **A1.**
*A message $M\in \{1,\ldots ,2^{lR}\}$ is stored on n nodes, of which at least arbitrary k is accessed for decoding. With $lR^{\prime }>\frac{1}{k}lR$ bits on each node, decoding is possible with error P(E)→0 as l→∞.*

### Appendix A.1. Codebook Generation

The codebooks $C_{I_{n-2}I_{n}}$ are generated and binned exactly as in [4]. The difference from [4] is that there is no *n*-th layer, and that at layer n−1 there is only one codebook $C_{n-1}$. The codebook $C_{n-1}$ of size $2^{lR_{n-1}^{\prime }}$ is generated like $C_{n}$ in [4], but then stored on the nodes with an (n,n−1) MDS code.

We also generate n−2 common codebooks $C_{uI_{n-2}}$ by drawing $2^{lR_{uk}^{\prime }}$ codewords $\left(u_{k}^{\left(l\right)}\left(1\right),\ldots ,u_{k}^{\left(l\right)}\left(2^{lR_{u_{k}}^{\prime }}\right)\right)$ independently with replacement over the set $T_{\epsilon }^{l}\left(U_{k}\right)$ according to a uniform distribution. The indices for $C_{uk},k=2,\ldots ,,n-2$ are next binned. Let $\xi_{uk}=2^{l(R_{uk}^{\prime }-R_{uk}+\gamma_{uk})}$ for some $\gamma_{k}>0$ and make $2^{lR_{uk}}$ bins. For each bin, select $\xi_{uk}$ numbers from the set $\left\{1,\ldots ,2^{lR_{uk}^{\prime }}\right\}$, uniformly and with replacement. They are finally coded with an (n,k) MDS erasure code.

We finally generate (n−1) repair codebooks through binning. First, if

(A1) $$\begin{array}{cc} 0 & >H(Y_{kn}|U_{I_{k}},Y_{kI_{n-1}}Y_{I_{k-1}I_{n}})\\ \; & -\frac{1}{n}H\left(Y_{kI_{n}}|X,Y_{k-1I_{n}},U_{I_{k}}\right) \end{array}$$

it turns out, as will be seen later, that the lost codeword can be recovered from the remaining ones with high probability. In that case, we set $R_{rk}=0$ and store no extra repair information. For consistency, we think of there being one bin at layer *k* containing all codewords. Otherwise, we let $\xi_{rk}=2^{l(nR_{k}^{\prime }-R_{rk}+\gamma_{rk})}$ for some $\gamma_{rk}>0$ and make $2^{lR_{rk}}$ bins. For each bin, select $\xi_{rk}$ vectors from the set ${\left\{1,\ldots ,2^{lR_{k}^{\prime }}\right\}}^{n}$, uniformly and with replacement. The bin indices are further coded with an (n,n−1) MDS erasure code.

### Appendix A.2. Encoding

Given a source codeword $x^{\left(l\right)}\in X^{l}$, we find codewords so that

$$\left(x^{\left(l\right)},u_{I_{n-2}}^{\left(l\right)}\left(V_{I_{n-2}}\right),y_{I_{n-2}I_{n}}^{\left(l\right)}.*\left(Q_{I_{n-2}I_{m}}\right),y_{n-1}^{\left(l\right)}\left(Q_{n-1}\right)\right)$$

are jointly typical. The binning of $Q_{I_{n-2}I_{m}}$ and $Q_{n-1}$ are done exactly as in [4] to obtain bin indices $B_{I_{n-2}I_{n}},B_{n-1}$. The bin index $B_{n-1}$ is further coded with the (n,n−1) MDS code. For $V_{k}$, we find the smallest bin index $B_{uk}$ that contains $V_{k}$ (if $V_{k}$ is in no bin, $B_{uk}=0$), and this is further coded with the (n,k) MDS code.

For repair, for those $k\in I_{n-1}$ where repair information is needed, we find the smallest bin index $W_{k}$ so that $Q_{kI_{n}}$ is in the corresponding bin; if no bin contains $W_{k}$, we put $W_{k}=0$. These are then coded with the (n,n−1) MDS code.

### Appendix A.3. Decoding

We assume node 1,2,…,j are available. The bin indices $B_{uI_{j^{\prime }}}$ are decoded from the MDS code, where $j^{\prime }=\min\left\{j,n-2\right\}$. The decoding now is similar to [4], except that there is also a common codeword. Consider decoding at layer $k\in \{2,\ldots ,j^{\prime }\}$. First, we find an index $V_{k}$ in bin $B_{uk}$ so that

$$\left(y_{I_{k-1}I_{j}}^{\left(l\right)},u_{k}^{\left(l\right)}\left(V_{k}\right),u_{I_{k-1}}^{\left(l\right)}\right)\in T_{\epsilon }^{l}\left(Y_{I_{k-1}I_{j}},U_{I_{k}}\right)$$

Next, for any size *k* subset $S\subset I_{j}$, the decoder looks in bins $B_{kS}$ for codewords $y_{kS}^{\left(l\right)}$ so that

$$\left(y_{kS}^{\left(l\right)},y_{I_{k-1}I_{j}}^{\left(l\right)},u_{I_{k}}^{\left(l\right)}\right)\in T_{\epsilon }^{l}\left(Y_{kI_{k}},Y_{I_{k-1}I_{j}},U_{I_{k}}\right)$$

If j=n−1, $B_{n-1}$ is first recovered from the MDS code. Then, the above procedure is repeated (there is no $U_{n-1}$).

The reconstructions of ${\hat{x}}^{\left(l\right)}$ are standard as in [4].

### Appendix A.4. Repair

Without loss of generality and to simplify notation, we can assume that node *n* fails. The repair is done layer by layer. At layer 1, we copy $V_{1}$ from any node to the replacement node *n*. Next, from the (n−1) surviving nodes we decode the repair bin index $W_{1}$ from the MDS code; if there is no extra repair information, we put $W_{1}=1$. We know $Q_{1I_{n-1}}$ from the surviving nodes. In bin $W_{1}$, we look for an index $Q_{1n}$ so that the corresponding codeword $\left(y^{\left(l\right)}.*\left(Q_{1I_{n}}\right),u_{1}^{\left(l\right)}\left(V_{1}\right)\right)\in T_{\epsilon }^{l}\left(Y_{1I_{n}},U_{1}\right)$; if there is more than one, there is a repair error. We then store the recovered $Q_{1n}$ in the replacement node *n*.

The following layers proceed in almost the same way. However, now to recover the common message $V_{k}$ we arbitrarily choose *k* of the surviving nodes and decode $V_{k}$ just as with usual operation. The decoded $V_{k}$ is then encoded with the exact same MDS code and we store the corresponding codeword on the replacement node *n*. We next find an index $Q_{kn}$ in bin $W_{k}$ so that $\left(y_{I_{k}I_{n}}^{\left(l\right)}.*\left(Q_{I_{k}I_{n}}\right),u_{I_{k}}^{\left(l\right)}\left(V_{I_{k}}\right)\right)\in T_{\epsilon }^{l}\left(Y_{I_{k}I_{n}},U_{I_{k}}\right)$.

On the last layer, we simply decode $Q_{n-1}$ from the surviving nodes as usual, and then we re-encode with the same MDS code, and store the recovered bin index on the new node *n*.

We notice that this repair is exact: the information on the restored node is exactly the same as on the failed node, except if a repair error happens.

### Appendix A.5. Analysis of Decoding Error

We have some slightly modified error events compared to [4] and some additional ones. We find it necessary to write these down explicitly

1. $E_{0}$: $x^{\left(l\right)}\notin T_{\epsilon }^{l}\left(X\right)$.
2. $E_{1}$: There exists no indices so that
  $$\begin{array}{c} \left(x^{\left(l\right)},u_{I_{n-2}}^{\left(l\right)}\left(V_{I_{n-2}}\right),y_{I_{n-2}I_{n}}^{\left(l\right)}.*\left(Q_{I_{n-2}I_{m}}\right),\right.\\ \left.y_{n-1}^{\left(l\right)}\left(Q_{n-1}\right)\right)\\ \in T_{\epsilon }^{l}\left(X,U_{I_{n-2}},Y_{I_{n-2}I_{n}},Y_{n-1}\right) \end{array}$$
3. $E_{2}$: Not all the indices $\left(B_{2I_{n}},\ldots ,B_{\left(n-2\right)I_{n}},B_{n-1}\right)$ are greater than zero.
4. $E_{3}$: For some subset $S\subset I_{n}$ with |S|=k∈{2,…,n−2} there exists some other $Q_{kS}^{\prime }$ in bins $B_{kS}$ so that (We use a slightly different notation for $E_{3}$ compared to [4], which we think is clearer.
  $$\left(y_{kS}^{\left(l\right)}\left(Q_{kS}^{\prime }\right),y_{I_{k-1}I_{j}}^{\left(l\right)},u_{I_{k}}^{\left(l\right)}\right)\in T_{\epsilon }^{l}\left(Y_{kI_{k}},Y_{I_{k-1}I_{k}},U_{I_{k}}\right)$$
5. $E_{4}$: Not all the indices $B_{uk}$ are greater than zero.
6. $E_{5}$: For some 2≤k≤n−2 there exist another index $V_{k}^{\prime }\neq V_{k}$ in bin $B_{uk}$ so that
  (A2) $$\left(y_{I_{k-1}I_{j}}^{\left(l\right)},u_{k}^{\left(l\right)}\left(V_{k}^{\prime }\right),u_{I_{k-1}}^{\left(l\right)}\right)\in T_{\epsilon }^{l}\left(Y_{I_{k-1}I_{k}},U_{I_{k}}\right)$$
7. $E_{6}$: There is a decoding error in the (n,k) MDS erasure code for $B_{uk}$.
8. $E_{7}$: There is a decoding error in the (n,n−1) MDS erasure code for $B_{n-1}$.

First by Remark A1, $P\left(E_{6}\right),P\left(E_{7}\right)\to 0$ as long as the rates before the MDS is scaled appropriately.

As in [4] we have $P(E_{0})\to 0$ as l→∞. For $E_{1}$ as in [4] we define $E_{1i}$ as an encoding error on layer *i* given that the previous layers have been encoded correctly and *in addition*, here, that $u_{i}^{\left(l\right)}$ has been encoded correctly. Then, as in [4], we find that $P(E_{1i})\to 0$ if

(A3) $$\begin{array}{cc} nR_{1}^{\prime } & >nH\left(Y_{11}\right)-H\left(Y_{1I_{n}}|X,U_{1}\right)\\ nR_{i}^{\prime } & >nH\left(Y_{i1}\right)-H\left(Y_{iI_{n}}|X,Y_{I_{i-1}I_{n}},U_{I_{i-1}}\right)\\ nR_{n-1}^{\prime } & >I(Y_{n-1};X,Y_{I_{n-2}I_{n}}U_{I_{n-2}}) \end{array}$$

with the difference being the addition of the $U_{*}$ variables. Similarly, we can define $E_{1i}^{u}$ as an encoding error of $u_{i}^{\left(l\right)}$ given that the previous layers have been encoded correctly, and we similarly have that $P(E_{1i}^{u})\to 0$ if

(A4) $$\begin{array}{cc} R_{u1}^{\prime } & >H\left(U_{1}\right)-H\left(U_{1}|X\right)\\ R_{ui}^{\prime } & >H\left(U_{i}\right)-H\left(U_{i}|X,Y_{I_{i-1}I_{n}},U_{I_{i-1}}\right) \end{array}$$

The proof that $P(E_{2})\to 0$ is unchanged from [4], and the proof that $P(E_{4})\to 0$ is similar.

The proof that $P(E_{3})\to 0$ is similar to [4], except that at the time of decoding at layer *k* the decoder has access to $u_{I_{k}}^{\left(l\right)}$. The relevant probability of decoding error at layer *k* is therefore $P(E_{3k}|E_{3I_{k-1}}^{c},E_{2}^{c},E_{4}^{c},E_{5I_{k}}^{c},E_{6}^{c},E_{7}^{c})$, and since we search for codewords in $T_{\epsilon }^{l}\left(Y_{kI_{k}},Y_{I_{k-1}I_{j}},U_{I_{k}}\right)$, the condition for this error probability converging to zero is

(A5) $$R_{k}>R_{k}^{\prime }-H\left(Y_{k1}\right)+\frac{1}{k}H\left(Y_{kI_{k}}|U_{I_{k}},Y_{I_{k-1}I_{k}}\right)$$

instead of [4] (A17).

To prove that $P(E_{5})\to 0$, we let $E_{5k}$ be the decoding error on layer *k*, and then bound $P_{5k}=P\left(E_{5k}|E_{3I_{k-1}}^{c},E_{2}^{c},E_{4}^{c},E_{5I_{k-1}}^{c},E_{6}^{c},E_{7}^{c}\right)$. If we pick a random codeword $u_{k}^{\left(l\right)}\in T_{\epsilon }^{l}\left(U_{k}\right)$, the probability that this is jointly typical, i.e., the event (A2), is

$$P\leq 2^{-l(I\left(U_{k};Y_{I_{k-1}}U_{I_{k-1}}\right)-\delta \left(\epsilon \right))}$$

There are $\xi_{uk}=2^{l(R_{uk}^{\prime }-R_{uk}+\gamma_{uk})}$ elements in each bin, and therefore,

$$P_{5k}\leq \xi_{uk}P$$

if we let $\gamma_{uk}>\delta \left(\epsilon \right)$, we have $P_{5k}\to 0$ if

$$R_{uk}^{\prime }-R_{uk}<I\left(U_{k};Y_{I_{k-1}}U_{I_{k-1}}\right)$$

Together with (A4), this gives (5).

### Appendix A.6. Analysis of Repair Error

If $E_{4}-E_{7}$, from above happen, there is also a repair error. Notice that at time of repair, we have access to n−1 nodes, and we can therefore use decoding for n−1 nodes, and in that case we have proven that $\sum_{i=4}^{7}P\left(E_{i}\right)\to 0$ as l→∞. We have the following additional repair error events:

1. $E_{r1}$: Some $W_{k}=0$ for $k\in I_{n-2}$.
2. $E_{r2}$: For $k\in I_{n-2}$, there exists another bin index $Q_{kn}^{\prime }$ in bin $W_{k}$ so that
  $$\begin{array}{c} \left(y_{kI_{n}}^{\left(l\right)}\left(Q_{kI_{n}}^{\prime }\right),y_{I_{k-1}I_{n}}^{\left(l\right)}.*\left(Q_{I_{k-1}I_{n}}\right),u_{I_{k}}^{\left(l\right)}\left(V_{I_{k}}\right)\right)\\ \in T_{\epsilon }^{l}\left(Y_{I_{k}I_{n}},U_{I_{k}}\right) \end{array}$$
3. $E_{r3}$: For $k\in I_{n-2}$, there is a decoding error in the (n,n−1) MDS erasure code for $W_{k}$.

#### Appendix A.6.1. Bounding *E**_r_*_1_

In total, for all bins, we pick $N=2^{lR_{rk}}\xi_{rk}=2^{l(nR_{k}^{\prime }+\gamma_{rk})}$ elements with replacement from a set of size $2^{nlR_{rk}^{\prime }}$. The probability that a particular element was never picked is then $P\left(E_{r1}\right)={\left(1-2^{-nlR_{rk}^{\prime }}\right)}^{N}$ and

$$\begin{array}{cc} \log P(E_{r1}) & =N\log\left(1-2^{-nlR_{rk}^{\prime }}\right)\leq -N2^{-nlR_{rk}^{\prime }}\\ \; & =-2^{l\gamma_{rk}}\to -\infty \;\mathrm{as}\;l\to \infty \end{array}$$

#### Appendix A.6.2. Bounding *E_r_*_2_

First, we will argue that if (A1) is satisfied, we can predict $y_{kn}^{\left(l\right)}$ with probability approaching one. We can state this as follows: if we pick a random $y_{kn}^{\left(l\right)}\in T_{\epsilon }^{l}\left(Y_{kn}\right)$, what is the probability *P* that

(A6) $$\begin{array}{c} \left(y_{kn}^{\left(l\right)},y_{kI_{n-1}}^{\left(l\right)}\left(Q_{kI_{n-1}}\right),y_{I_{k-1}I_{n}}^{\left(l\right)}.*\left(Q_{I_{k-1}I_{n}}\right),u_{I_{k}}^{\left(l\right)}\left(V_{I_{k}}\right)\right)\\ \in T_{\epsilon }^{l}\left(Y_{I_{k}I_{n}},U_{I_{k}}\right) \end{array}$$

This is actually a standard channel coding problem, so we get

(A7) $$P\leq 2^{-l(I\left(Y_{kn};Y_{kI_{n-1}},Y_{iI_{n-1}},U_{I_{k}}\right)-\delta \left(\epsilon \right))}$$

Since the codebook $C_{uk}$ has $2^{lR_{k}^{\prime }}$ elements, we then have

$$P\left(E_{r2k}\right)\leq 2^{lR_{k}^{\prime }}P$$

Thus, $P(E_{r2k})\to 0$ as l→∞ if

$$R_{k}^{\prime }<H\left(Y_{kn}\right)-H\left(Y_{kn}|Y_{kI_{n-1}},Y_{iI_{n-1}},U_{I_{k}}\right)-\delta \left(\epsilon \right)$$

Now in consideration of (A5) there is no gain from making $R_{k}^{\prime }$ larger than needed. Thus, $R_{k}^{\prime }$ is chosen arbitrarily close to the limit given by (A3), and we therefore have $P(E_{r2k})\to 0$ if

$$\begin{array}{cc} \; & H\left(Y_{k1}\right)-\frac{1}{n}H\left(Y_{kI_{n}}|X,Y_{I_{k-1}I_{n}},U_{I_{k-1}}\right)\\ \; & <H\left(Y_{kn}\right)-H\left(Y_{kn}|Y_{kI_{n-1}},Y_{iI_{n-1}},U_{I_{k}}\right)-\delta \left(\epsilon \right) \end{array}$$

which is (A1).

Now, turn to the case when (A1) is not satisfied. We look for vectors $\left(Q_{k1}^{\prime },Q_{k2}^{\prime },\ldots ,Q_{kn}^{\prime }\right)\in {\left\{1,\ldots ,2^{lR_{k}^{\prime }}\right\}}^{n}$ that

1. Are in the bin indicated by $W_{k}$.
2. Has $Q_{ki}^{\prime }=Q_{ki}$, i≤n−1.
3. Are jointly typical, i.e., satisfy (A6).

For condition 3, (A7) is still valid. Each bin contains $\xi_{rk}=2^{l(nR_{k}^{\prime }-R_{rk}+\gamma_{rk})}$ vectors. Each of these has probability $P_{2}=2^{-l\left(n-1\right)R_{lk}}$ of satisfying conditions 2. Therefore,

$$P\left(E_{r2k}\right)\leq \xi_{rk}P_{2}P=2^{l(R_{k}^{\prime }-R_{rk}+\gamma_{rk})}P$$

if we choose $\gamma_{rk}>\delta \left(\epsilon \right)$ we have $P(E_{r2k})\to 0$ as l→∞ if

$$R_{k}^{\prime }-R_{rk}<H\left(Y_{kn}\right)-H\left(Y_{kn}|Y_{kI_{n-1}},Y_{iI_{n-1}},U_{I_{k}}\right)$$

Which together with (A3) and the argument above leads to (6).

## Appendix B. Proof of Theorem 3

We use the following simple converse: when one node fails and the remaining n−1 nodes collaborates with the repair node, they have to be able to restore *X* with distortion $D_{n}$. Therefore,

(A8) $$\left(n-1\right)R+R_{r}\geq \frac{1}{2}\log\left(\frac{1}{D_{n}}\right)$$

While the calculations in the proof are in principle straightforward, we include some detail to make it simpler for readers to further develop the results. The three different cases in the Theorem are as in [9] (Section VI.A). We put

$$\begin{array}{cc} Y_{kI_{n}} & =X+Q_{k}\\ U_{k} & =X+Q_{u}\\ Y_{n} & =X+Q_{n} \end{array}$$

with $Q_{\ldots }$ zero-mean Gaussian, $E\left[Q_{u}^{2}\right]=\sigma_{u}^{2}$, $E\left[Q_{ki}^{2}\right]=\sigma_{k}^{2}$, $E\left[Q_{n}^{2}\right]=\sigma_{n}^{2}$, $E\left[Q_{ki}Q_{kj}\right]=\rho \sigma_{k}^{2}$ for i≠j, and all other noise variables uncorrelated. Let

$$\begin{array}{cc} R_{k} & =\left(1-\rho \right)I+\rho 11^{T}\\ R_{k}^{-1} & =\frac{1}{1-\rho }I-\frac{\rho }{\left(1-\rho \right)\left(1+(k-1)\rho \right)}11^{T} \end{array}$$

Here, 1 is a column vector of all 1s, so $11^{T}$ is a matrix of all ones.

We first calculate the distortions,

(A9) $$\begin{array}{cc} D_{k} & =\left[\begin{array}{cc} \sigma_{u}^{2} & 0\\ 0 & \sigma_{k}^{2}R_{k} \end{array}\right]\\ D_{k} & ={\left(1+1^{T}D_{k}^{-1}1\right)}^{-1}\\ & ={\left(1+\sigma_{u}^{-2}+\sigma_{k}^{-2}\left(\frac{1}{1-\rho }k-\frac{\rho k^{2}}{\left(1-\rho \right)\left(1+(k-1)\rho \right)}\right)\right)}^{-1} \end{array}$$

(A10) $$\begin{array}{c} ={\left(1+\sigma_{u}^{-2}+\frac{k\sigma_{k}^{-2}}{1+(k-1)\rho }\right)}^{-1} \end{array}$$

(A11) $$\begin{array}{cc} & =\frac{\left(1+\left(k-1\right)\rho \right)\sigma_{k}^{2}\sigma_{u}^{2}}{\left(1+\left(k-1\right)\rho \right)\sigma_{k}^{2}\sigma_{u}^{2}+\left(1+\left(k-1\right)\rho \right)\sigma_{k}^{2}+k\sigma_{u}^{2}} \end{array}$$

(A12) $$\begin{array}{cc} Q_{2} & =\left[\begin{array}{ccc} \sigma_{n}^{2} & 0 & 0\\ 0 & \sigma_{u}^{2} & 0\\ 0 & 0 & \sigma_{k}^{2}R_{n} \end{array}\right]\\ D_{n} & ={\left(1+1^{T}Q_{2}^{-1}1\right)}^{-1}\\ & ={\left(1+\sigma_{n}^{-2}+\sigma_{u}^{-2}+\frac{n\sigma_{k}^{-2}}{1+(n-1)\rho }\right)}^{-1} \end{array}$$

The $D_{k}$ distortion constraint is always satisfied with equality, and therefore

(A13) $$\sigma_{k}^{2}=\frac{kD_{k}\sigma_{u}^{2}}{\left(1+\left(k-1\right)\rho \right)\left(\sigma_{u}^{2}-D_{k}\sigma_{u}^{2}-D_{k}\right)}$$

In general, we can write

$$\begin{array}{cc} h(X|Y) & =\frac{1}{2}\log\left({\left(2\pi e\right)}^{n}\det K_{X|Y}\right)\\ \; & =\frac{1}{2}\log\left({\left(2\pi e\right)}^{n}\det\left(K_{\mathrm{XX}}-K_{\mathrm{YX}}^{T}K_{\mathrm{YY}}^{-1}K_{\mathrm{YX}}\right)\right) \end{array}$$

So we just need to the various conditional covariances

$$\begin{array}{cc} K_{Y_{kI_{k}}|U_{k}} & =11^{T}+\sigma_{k}^{2}R_{k}-\frac{1}{1+\sigma_{u1}^{2}}11^{T}=\sigma_{k}^{2}R_{k}+\frac{\sigma_{u1}^{2}}{1+\sigma_{u1}^{2}}11^{T}\\ K_{Y_{kI_{n}},U_{k}} & =11^{T}+\left[\begin{array}{cc} \sigma_{u}^{2} & 0\\ 0 & \sigma_{k}^{2}R_{n} \end{array}\right]\\ K_{Y_{kI_{n}},U_{k}}^{-1} & =\left[\begin{array}{cc} \sigma_{u}^{-2} & 0\\ 0 & \sigma_{k}^{-2}R_{n}^{-1} \end{array}\right]-\frac{\left[\begin{array}{cc} \sigma_{u}^{-2} & 0\\ 0 & \sigma_{k}^{-2}R_{n}^{-1} \end{array}\right]11^{T}\left[\begin{array}{cc} \sigma_{u}^{-2} & 0\\ 0 & \sigma_{k}^{-2}R_{n}^{-1} \end{array}\right]}{1+1^{T}\left[\begin{array}{cc} \sigma_{u}^{-2} & 0\\ 0 & \sigma_{k}^{-2}R_{n}^{-1} \end{array}\right]1}\\ K_{Y_{n}|Y_{kI_{n}}U_{k}} & =1+\sigma_{n}^{2}-1^{T}K_{Y_{kI_{n}},U_{k}}^{-1}1\\ \; & =1+\sigma_{n}^{2}-\frac{\left(1+(n-1)\rho \right)\sigma_{k}^{2}+n\sigma_{u}^{2}}{\left(1+(n-1)\rho \right)\sigma_{k}^{2}\left(\sigma_{u}^{2}+1\right)+n\sigma_{u}^{2}}\\ K_{Y_{kI_{n}}|XU} & =K_{Q_{k}}=\sigma_{k}^{2}R_{n}\\ K_{Y_{n}|XU_{k}} & =\sigma_{n}^{2} \end{array}$$

We need

$$\begin{array}{cc} \det\left(K_{Y_{kI_{k}}|U_{k}}\right) & =\det\left(\sigma_{k}^{2}R_{k}+\frac{\sigma_{u}^{2}}{1+\sigma_{u}^{2}}11^{T}\right)\\ \; & =\left(1-\frac{\sigma_{k}^{-2}\sigma_{u}^{2}}{1+\sigma_{u}^{2}}1^{T}R_{k}^{-1}1\right)\det\sigma_{k}^{2}R_{k}\\ \; & =\left(1+\frac{\sigma_{u}^{2}}{1+\sigma_{u}^{2}}\frac{k\sigma_{k}^{-2}}{1+(k-1)\rho }\right)\sigma_{k}^{2k}\left(1+\frac{\rho }{1-\rho }1^{T}I1\right)\det\left(1-\rho \right)I\\ \; & =\left(1+\frac{\sigma_{u}^{2}}{1+\sigma_{u}^{2}}\frac{k\sigma_{k}^{-2}}{1+(k-1)\rho }\right){\left(\left(1-\rho \right)\sigma_{k}^{2}\right)}^{k}\left(1+\frac{k\rho }{1-\rho }\right)\\ \det\left(K_{Y_{kI_{n}}|XU_{k}}\right) & =\det\left(\sigma_{k}^{2}R_{n}\right)\\ \; & ={\left(\left(1-\rho \right)\sigma_{k}^{2}\right)}^{n}\left(1+\frac{n\rho }{1-\rho }\right) \end{array}$$

Then we get

$$\begin{array}{cc} R & =\frac{1}{2k}\log\left(\left(1-\frac{1}{1+\sigma_{u1}^{2}}\frac{k\sigma_{k}^{-2}}{1+(k-1)\rho }\right){\left(\left(1-\rho \right)\sigma_{k}^{2}\right)}^{k}\left(1+\frac{k\rho }{1-\rho }\right)\right)\\ \; & +\frac{1}{2n}\log\left(\frac{1}{\sigma_{n}^{2}}\left(1+\sigma_{n}^{2}-\frac{\left(1+(n-1)\rho \right)\sigma_{k}^{2}+n\sigma_{u}^{2}}{\left(1+(n-1)\rho \right)\sigma_{k}^{2}\left(\sigma_{u}^{2}+1\right)+n\sigma_{u}^{2}}\right)\right)\\ \; & -\frac{1}{2n}\log\left({\left(\left(1-\rho \right)\sigma_{k}^{2}\right)}^{n}\left(1+\frac{n\rho }{1-\rho }\right)\right)\\ \; & +\frac{1}{2k}\log\left(1+\frac{1}{\sigma_{u}^{2}}\right) \end{array}$$

For repair we need

$$\begin{array}{cc} K_{U_{k}Y_{kI_{n-1}}} & =11^{T}+\left[\begin{array}{cc} \sigma_{u}^{2} & 0\\ 0 & \sigma_{k}^{2}R_{n-1} \end{array}\right]\\ K_{U_{k}Y_{kI_{n-1}}}^{-1} & =\left[\begin{array}{cc} \sigma_{u}^{-2} & 0\\ 0 & \sigma_{k}^{-2}R_{n-1}^{-1} \end{array}\right]-\frac{\left[\begin{array}{cc} \sigma_{u}^{-2} & 0\\ 0 & \sigma_{k}^{-2}R_{n-1}^{-1} \end{array}\right]11^{T}\left[\begin{array}{cc} \sigma_{u}^{-2} & 0\\ 0 & \sigma_{k}^{-2}R_{n-1}^{-1} \end{array}\right]}{1+1^{T}\left[\begin{array}{cc} \sigma_{u}^{-2} & 0\\ 0 & \sigma_{k}^{-2}R_{n-1}^{-1} \end{array}\right]1}\\ K_{Y_{kn},U_{k}Y_{kI_{n-1}}} & =\left[\begin{array}{cc} 1 & \left(1+\rho_{1}\sigma_{k}^{2}\right)1^{T} \end{array}\right]\\ K_{Y_{kI_{n}}|X,U_{k}} & =\sigma_{k}^{2}R_{n}\\ K_{Y_{kn}|U_{k}Y_{kI_{n-1}}} & =1+\sigma_{k}^{2}-\frac{\sigma_{u}^{-2}+\frac{\left(n-1\right)\sigma_{k}^{-2}}{1+(n-2)\rho }{\left(1+\rho_{1}\sigma_{k}^{2}\right)}^{2}}{1+\sigma_{u}^{-2}+\frac{\left(n-1\right)\sigma_{k}^{-2}}{1+(n-2)\rho }}\\ \; & =1+\sigma_{k}^{2}-\frac{\left(1+(n-2)\rho \right)\sigma_{k}^{2}+\left(n-1\right)\sigma_{u}^{2}{\left(1+\rho_{1}\sigma_{k}^{2}\right)}^{2}}{\left(1+(n-2)\rho \right)\sigma_{k}^{2}\left(\sigma_{u}^{2}+1\right)+\left(n-1\right)\sigma_{u}^{2}} \end{array}$$

From which

(A14) $$\begin{array}{cc} R_{r} & =\frac{1}{2}\log\left(1+\sigma_{k}^{2}-\frac{\left(1+(n-2)\rho \right)\sigma_{k}^{2}+\left(n-1\right)\sigma_{u}^{2}{\left(1+\rho_{1}\sigma_{k}^{2}\right)}^{2}}{\left(1+(n-2)\rho \right)\sigma_{k}^{2}\left(\sigma_{u}^{2}+1\right)+\left(n-1\right)\sigma_{u}^{2}}\right)\\ \; & -\frac{1}{2n}\log\left({\left(\left(1-\rho \right)\sigma_{k}^{2}\right)}^{n}\left(1+\frac{n\rho }{1-\rho }\right)\right)\\ \; & +\frac{1}{2n}\log\left(\frac{1}{\sigma_{n}^{2}}\left(1+\sigma_{n}^{2}-\frac{\left(1+(n-1)\rho \right)\sigma_{k}^{2}+n\sigma_{u}^{2}}{\left(1+(n-1)\rho \right)\sigma_{k}^{2}\left(\sigma_{u}^{2}+1\right)+n\sigma_{u}^{2}}\right)\right) \end{array}$$

For case 1, we set $\sigma_{n}^{2}=\infty$ and ρ=0. Then

$$R=\frac{1}{2k}\log\left(\frac{1}{D_{k}}\right)$$

independent of $\sigma_{u}^{2}$. We choose $\sigma_{u}^{2}$ so that we get exactly $D_{n}$. Solving for $\sigma_{u}^{2}$ and inserting in (A14) results in

$$R_{r}=\frac{1}{2}\log\left(\frac{D_{k}\left(n-k\right)}{D_{k}\left(n-k-1\right)+D_{n}}\right)$$

Then,

$$\left(n-1\right)R+R_{r}=\frac{1}{2}\log\left(\frac{D_{k}\left(n-k\right)}{D_{k}\left(n-k-1\right)+D_{n}}\frac{1}{D_{k}^{(n-1)/k}}\right)$$

for k=n−1 we get

$$\left(n-1\right)R+R_{r}=\frac{1}{2}\log\left(\frac{D_{k}}{D_{n}}\frac{1}{D_{k}}\right)=\frac{1}{2}\log\left(\frac{1}{D_{n}}\right)$$

which achieves (A8)

For case 2, we put $\sigma_{u}^{2}=\sigma_{n}^{2}=\infty$. We solve for ρ so that we exactly achieve $D_{n}$,

$$\rho =\frac{D_{k}\left(\left(n-k\right)D_{n}+k\right)-D_{n}n}{D_{k}\left(D_{n}n-k\left(D_{n}+n-1\right)\right)+D_{n}\left(k-1\right)n}$$

Giving

$$\begin{array}{cc} R & =\frac{1}{2}\log\left({\left(\frac{\left(D_{k}-1\right)D_{n}\left(k-n\right)}{k(D_{k}-D_{n})}\right)}^{-1/n}{\left(\frac{\left(D_{n}-1\right)\left(k-n\right)}{n(D_{k}-D_{n})}\right)}^{1/k}\right)\\ R_{r} & =\frac{1}{2}\log\left(\frac{\left(D_{k}-1\right)n\left(n-k\right){\left(\frac{\left(D_{k}-1\right)D_{n}\left(k-n\right)}{k(D_{k}-D_{n})}\right)}^{-1/n}}{k\left(-D_{k}n+D_{n}+n-1\right)+\left(D_{k}-1\right)\left(n-1\right)n}\right) \end{array}$$

Inserting k=n−1 and simplifying, it is seen that (A8) is achieved.

Now region III. We put $\sigma_{u}^{2}=\infty$, and find $\sigma_{k}^{2}$ and $\sigma_{n}^{2}$ to exactly satisfy $D_{k}$ and $D_{n}$. We minimize the resulting (large) expression with respect to ρ, giving $\rho =\frac{D_{k}-1}{D_{k}+k-1}$. This results in

$$R=R_{r}=\frac{1}{2}\log\left(\frac{1}{\sqrt[{n}]{D_{n}}}\right)$$
